# 固相萃取-超高效液相色谱-三重四极杆质谱法测定饮用水中13种卤代苯醌类消毒副产物

**DOI:** 10.3724/SP.J.1123.2022.12006

**Published:** 2023-06-08

**Authors:** Yuanyuan WANG, Lulu LI, Jia LÜ, Yongyan CHEN, Lan ZHANG

**Affiliations:** 中国疾病预防控制中心环境与人群健康重点实验室, 中国疾病预防控制中心环境与健康相关产品安全所, 北京 100050; China CDC Key Laboratory of Environment and Population Health, National Institute of Environmental Health, Chinese Center for Disease Control and Prevention, Beijing 100050, China

**Keywords:** 固相萃取, 超高效液相色谱-三重四极杆质谱, 卤代苯醌, 消毒副产物, 饮用水, solid phase extraction (SPE), ultra performance liquid chromatography-triple quadrupole mass spectrometry (UPLC-MS/MS), halobenzoquinones (HBQs), disinfection by-products (DBPs), drinking water

## Abstract

卤代苯醌作为一类新检出的消毒副产物,在饮用水中检出率高但含量较低。为准确、高效、高通量分析饮用水中的卤代苯醌,本文基于固相萃取前处理和超高效液相色谱-三重四极杆质谱,建立了同时检测饮用水中13种卤代苯醌(6种氯代苯醌、6种溴代苯醌、1种碘代苯醌)的方法。在1 L水样中加入2.5 mL甲酸混匀,取500 mL水样经Plexa固相萃取柱(200 mg/6 mL)富集浓缩后,进行超高效液相色谱-三重四极杆质谱检测。以HSS T_3_色谱柱(100 mm×2.1 mm, 1.8 μm)分离,甲醇-0.1%甲酸水溶液为流动相进行梯度洗脱,采用电喷雾负离子模式电离、多反应监测模式检测,基质匹配外标法定量。以饮用水为基质考察方法的精密度和准确度,结果表明,13种卤代苯醌在各自的线性范围内呈现良好的线性关系,相关系数(*r*)均大于0.999,方法检出限(MDL, *S/N*=3)和方法定量限(MQL, *S/N*=10)分别为0.2~10.0 ng/L和0.6~33.0 ng/L。不同加标水平(10、20、50 ng/L)下13种卤代苯醌的回收率为56%~88%,相对标准偏差(RSD, *n*=6)均≤9.2%。利用该方法分析了5份实际饮用水样品,共检出4种卤代苯醌,分别是2,6-二氯-1,4-苯醌、2,5-二溴-1,4-苯醌、2,6-二溴-1,4-苯醌和2,6-二溴-3,5-二甲基-1,4-苯醌。单一样品中若以任一卤代苯醌检出为标准,则卤代苯醌总检出率为100%。其中2,6-二氯-1,4-苯醌的含量最高,为15.0~56.2 ng/L。本方法具有良好的灵敏度、准确度和精密度,分析时间短,覆盖目标物种类多,适合饮用水中卤代苯醌类消毒副产物的测定,同时为研究饮用水中卤代苯醌的分布特征、健康风险及控制措施提供了有力支撑。

## Abstract (en)

Disinfection of drinking water is critical to prevent waterborne diseases. An unexpected consequence of water disinfection is the formation of disinfection by-products by the interaction of disinfectants with organic matter (natural or anthropogenic) and halides, which present significant toxicological effects and carcinogenic risks. As an emerging disinfection by-product, halobenzoquinones (HBQs) have attracted increasing attention owing to their severe toxicity and high detection rates. The credible determination of HBQs is essential for further studies on their occurrence, toxicity, and control measures; however, HBQs are usually detected in drinking water at trace levels. Therefore, accurate and efficient analytical techniques are critical for HBQ determination and quantitation. In this study, a method based on solid phase extraction (SPE) combined with ultra performance liquid chromatography-triple quadrupole mass spectrometry (UPLC-MS/MS) was developed to determine 13 HBQs, including six chlorobenzoquinones, six bromobenzoquinones, and one iodobenzoquinone, in drinking water. One-liter water samples were added with 2.5 mL of formic acid, and 500 mL of each sample was collected for further enrichment. Pretreatment optimization mainly focused on the SPE column, washing solvent, and nitrogen blowing temperature. After extraction using Plexa SPE columns (200 mg/6 mL), the samples were washed with ultrapure water containing 0.25% formic acid combined with 30% methanol aqueous solution containing 0.25% formic acid, eluted with 6 mL of methanol containing 0.25% formic acid, and then nitrogen blown at 30 ℃. The UPLC-MS/MS parameters were optimized by comparing the results of two reversed-phase columns (BEH C_18_ and HSS T_3_) and various concentrations of formic acid in the mobile phase, as well as by establishing the best instrumental conditions. The separation of 13 HBQs was performed using an HSS T_3_ column (100 mm×2.1 mm, 1.8 μm) via gradient elution with a mixture of 0.1% formic acid aqueous solution and methanol as the mobile phase for 16 min. The 13 HBQs were detected using a triple quadrupole mass spectrometer equipped with a negative electrospray ionization source (ESI^-^) in multiple reaction monitoring (MRM) mode. Matrix-matched calibration curves were used to quantify the HBQs owing to intense matrix inhibitory effects. The results reflected the good linear relationships of the 13 HBQs and yielded correlation coefficients (*r*) greater than 0.999. The method detection limits (MDLs, *S/N*=3) were 0.2-10.0 ng/L, while the method quantification limits (MQLs, *S/N*=10) were 0.6-33.0 ng/L. The recoveries of the 13 HBQs were 56%-88% at three spiked levels (10, 20, 50 ng/L), and the relative standard deviations (RSDs, *n*=6) were less than or equal to 9.2%. The optimization method was applied to analyze HBQs in five drinking water samples. Four HBQs, namely, 2,6-dichloro-1,4-benzoquinone (2,6-DCBQ), 2,5-dibromo-1,4-benzoquinone (2,5-DBBQ), 2,6-dibromo-1,4-benzoquinone (2,6-DBBQ), and 2,6-dibromo-3,5-dimethyl-1,4-benzoquinone (2,6-DBDMBQ), were detected in the samples with detection rates of 100%, 20%, 80%, and 20%, respectively. The most frequently detected HBQ, 2,6-DCBQ, also exhibited the highest content (15.0-56.2 ng/L). The method showed high sensitivity, stability, accuracy, and efficiency, rendering it suitable for the analysis of 13 HBQs in drinking water. Compared with previous methods that mainly focused on 2,6-DCBQ and 2,6-DBBQ, the developed method achieved higher throughput and enabled the simultaneous analysis of 13 HBQs. The method presented in this study provides an opportunity to explore different types and concentrations of HBQs in drinking water, offers a deeper understanding of the occurrence of HBQs, and facilitates further studies on the health risks and control measures of these compounds.

现代饮用水消毒处理工艺大大降低了介水流行病的传播风险。然而,消毒剂与水中的天然有机物、人为有机污染物、溴、碘化物等反应产生消毒副产物(DBPs),给环境和人类带来潜在危害^[1,2]^。卤代苯醌(halobenzoquinones, HBQs)是近年来发现的一类新检出的未受控消毒副产物,在加拿大^[3,4]^、美国^[4,5]^、日本^[6]^、中国^[7,8]^等各地水厂均有检出。定量结构-毒性效应(QSTR)分析显示,HBQs具有潜在致癌性,其毒性可能是受控消毒副产物卤乙酸、三氯甲烷^[9]^的1000倍以上^[10]^。体外实验表明,HBQs可诱导细胞产生大量活性氧,抑制谷胱甘肽生成,影响细胞抗氧化酶活性,从而导致细胞的氧化损伤^[11,12]^。基因毒性研究发现HBQs可与DNA直接或间接结合形成DNA加合物,影响基因组甲基化,导致DNA损伤和染色体异常^[13,14]^。以斑马鱼胚胎为模型探究发现,HBQs可引起斑马鱼氧化损伤和发育毒性,且半致死浓度(LC_50_)值比卤乙酸低约200倍^[15]^。

由于HBQs的毒性远高于受控消毒副产物,精准分析饮用水消毒后生成的HBQs种类和含量,对饮用水中HBQs暴露的健康风险评估、生物效应归因以及饮用水水质安全保障都具有重要意义。现阶段水中HBQs的检测方法主要有气相色谱法(GC)^[16]^、高效液相色谱法(HPLC)^[17]^和超高效液相色谱-串联质谱法(UPLC-MS/MS)^[8,18]^等。这些方法中,GC需对目标物进行衍生化处理,步骤繁琐;HPLC的灵敏度和定性能力较弱,容易出现假阳性结果;UPLC-MS/MS结合了色谱和质谱技术的优点,样品适用范围广,前处理简单快速,方法灵敏度高且选择性好^[19]^。但目前文献中针对HBQs的UPLC-MS/MS方法多以检测二氯苯醌、二溴苯醌为主,存在覆盖目标物种类较少、分析耗时较长、灵敏度有限、目标物回收率参差不齐等问题,难以实现水中多种类HBQs的同时高效检测。

针对上述问题,本文采用固相萃取-超高效液相色谱-串联质谱技术(SPE-UPLC-MS/MS),通过优化前处理条件和色谱、质谱参数,实现了饮用水中13种HBQs(包括6种氯代苯醌、6种溴代苯醌、1种碘代苯醌)的同时检测。该方法覆盖目标物种类多,高效快速,灵敏度和准确度均较好,为全面准确研究饮用水中HBQs的分布特征、环境行为和暴露风险提供了技术支撑。

## 1 实验部分

### 1.1 仪器、试剂与材料

液相色谱-三重四极杆质谱系统:Exion LC超高效液相色谱搭载QTRAP 5500三重四极杆质谱仪(美国SCIEX公司); SB5200DTD超声波清洗机(宁波新芝公司); Bond Elut Plexa固相萃取小柱(200 mg/6 mL,美国Agilent公司);全自动固相萃取仪、恒温水浴氮气吹干仪(睿科仪器厦门有限公司); GM-0.5A隔膜真空泵(天津津腾实验设备公司); MINI-230V涡流振荡器(美国Talboys公司); AL204-IC电子天平(感量0.000 1 g,瑞士Mettler公司); Milli-Q Integral纯水仪(美国Millipore公司)。

甲醇(LC-MS级,美国Merck公司),甲醇、甲酸(分别为HPLC级、LC-MS级,美国Fisher Scientific公司),甲酸(优级纯,上海安谱实验科技股份有限公司),实验用水为超纯水(18.2 MΩ·cm)。

13种HBQs标准物质:2,5-二氯-1,4-苯醌(2,5-DCBQ,纯度98%)、3,4,5,6-四氯-1,2-苯醌(TC-1,2-BQ,纯度97%)、3,4,5,6-四溴-1,2-苯醌(TB-1,2-BQ,纯度97%)、2,3-二溴-5,6-二甲基-1,4-苯醌(2,3-DBDMBQ,纯度99.9%)、2,3,5,6-四溴-1,4-苯醌(TBBQ,纯度98%)购于美国Sigma公司;2,6-二氯-1,4-苯醌(2,6-DCBQ,纯度98%)、2,5-二溴-1,4-苯醌(2,5-DBBQ,纯度95.2%)、2,6-二溴-3,5-二甲基-1,4-苯醌(2,6-DBDMBQ,纯度98%)购买于美国AccuStandard公司;2,3,6-三氯-1,4-苯醌(TriCBQ,纯度98%)、2,6-二氯-3-甲基-1,4-苯醌(DCMBQ,纯度98%)、2,6-二碘-1,4-苯醌(2,6-DIBQ,纯度98%)购买于上海艾康睿医药科技有限公司;2,6-二溴-1,4-苯醌(2,6-DBBQ,纯度98%)购买于加拿大TRC公司;2,3,5,6-四氯-1,4-苯醌(TCBQ,纯度99.9%)购买于德国Dr. Ehrenstorfer公司。

### 1.2 基质匹配混合标准溶液的配制

分别称取10.0 mg HBQs标准物质于10 mL棕色容量瓶中,用甲醇溶解并定容,配制成质量浓度均为1000 mg/L的13种HBQs标准储备液,转移至棕色试剂瓶,-20 ℃储存备用。准确移取各HBQs标准储备液200 μL于10 mL棕色容量瓶中,用甲醇定容,得到20 mg/L混合标准中间液,转移至棕色试剂瓶,-20 ℃储存备用。临用前,取空白水样经过与样品相同的前处理得到空白样品基质溶液,移取适量的HBQs混合标准中间液,用空白样品基质溶液稀释成质量浓度为0.2、0.5、1.0、2.0、5.0、10.0、20.0、40.0、50.0、100.0、200.0 μg/L的基质匹配系列混合标准溶液,以HBQs的峰面积(*y*)对质量浓度(*x*)绘制基质匹配工作曲线并计算相关系数(*r*)。

### 1.3 样品采集与前处理

用1 L棕色玻璃瓶采集水样,采集后立即加入2.5 mL甲酸,混匀。甲酸一方面可终止前体物与余氯继续反应,确保HBQs含量的真实性;另一方面可调节水样至酸性,保证HBQs的稳定性^[5]^。使用Plexa固相萃取小柱进行前处理富集:上样前分别用6 mL甲醇(含0.25%甲酸)和12 mL超纯水(含0.25%甲酸)活化固相萃取小柱。500 mL水样以6 mL/min的速度上样后,分别用6 mL超纯水(含0.25%甲酸)和6 mL 30%甲醇水溶液(含0.25%甲酸)进行淋洗,淋洗后用氮气吹干萃取小柱,用6 mL甲醇(含0.25%甲酸)洗脱。洗脱液在30 ℃下用氮气平缓吹至0.1 mL,用超纯水(含0.25%甲酸)定容至0.5 mL,涡旋混匀后使用UPLC-MS/MS进行检测。

### 1.4 分析条件

#### 1.4.1 色谱条件

色谱柱:Waters ACQUITY UPLC HSS T_3_(100 mm×2.1 mm, 1.8 μm),柱温:40 ℃;流动相A为0.1%甲酸水溶液,B为甲醇,流速为0.35 mL/min;进样体积:10 μL。梯度洗脱程序:0~8 min,流动相B由20%升至50%; 8~11 min,流动相B升至95%,保持2 min; 13.1 min时,流动相B快速降至20%并保持2.9 min。16 min内完成13种HBQs的分离检测。

#### 1.4.2 质谱条件

电喷雾离子源(ESI),负离子模式扫描,多反应监测(MRM)模式分析;离子源温度:700 ℃;气帘气:151 kPa;离子化气压:-4500 V;喷雾气:379 kPa;辅助加热气:448 kPa;碰撞气:62 kPa。其余质谱检测参数见表1。

**表1:** 13种HBQs的质谱参数及保留时间

Compound	Ion pair (m/z)		Retention time/min	DP/V	CE/eV	EP/V	CXP/V
2,5-Dichloro-1,4-benzoquinone (2,5-DCBQ)	177.0>	35.0	5.19±0.01	-70	-40	-10	-6
	177.0>	113.1^*^		-70	-40	-10	-6
2,6-Dichloro-1,4-benzoquinone (2,6-DCBQ)	177.0>	35.0	5.35±0.01	-70	-40	-10	-6
	177.0>	113.1^*^		-70	-40	-10	-6
2,5-Dibromo-1,4-benzoquinone (2,5-DBBQ)	266.9>	79.0	6.20±0.01	-60	-50	-10	-10
	266.9>	81.0^*^		-60	-50	-10	-10
2,6-Dibromo-1,4-benzoquinone (2,6-DBBQ)	266.9>	79.0	6.52±0.02	-60	-50	-10	-10
	266.9>	81.0^*^		-60	-50	-10	-10
2,6-Dichloro-3-methyl-1,4-benzoquinone (DCMBQ)	190.0>	35.0	7.73±0.01	-90	-43	-10	-6
	191.0>	126.9^*^		-90	-24	-10	-6
2,3,6-Trichloro-1,4-benzoquinone (TriCBQ)	210.9>	35.0	7.90±0.01	-100	-50	-10	-10
	210.9>	175.0^*^		-70	-20	-10	-10
2,6-Diiodo-1,4-benzoquinone (2,6-DIBQ)	360.0>	127.0^*^	8.19±0.01	-70	-33	-10	-10
	361.0>	127.0		-70	-45	-10	-10
2,3,5,6-Tetrachloro-1,4-benzoquinone (TCBQ)	245.0>	208.9^*^	9.98±0.01	-50	-20	-10	-10
	247.0>	210.9		-70	-25	-10	-10
2,6-Dibromo-3,5-dimethyl-1,4-benzoquinone (2,6-DBDMBQ)	293.9>	78.9	10.00±0.01	-60	-50	-10	-10
	293.9>	80.9^*^		-60	-50	-10	-10
2,3-Dibromo-5,6-dimethyl-1,4-benzoquinone (2,3-DBDMBQ)	293.9>	78.9	10.22±0.01	-60	-50	-10	-10
	293.9>	80.9^*^		-60	-50	-10	-10
2,3,5,6-Tetrabromo-1,4-benzoquinone (TBBQ)	424.7>	79.0	10.54±0.02	-70	-74	-10	-10
	424.7>	81.0^*^		-74	-74	-10	-10
3,4,5,6-Tetrachloro-1,2-benzoquinone (TC-1,2-BQ)	245.0>	35.0^*^	11.45±0.01	-50	-50	-10	-6
	247.0>	35.0		-50	-50	-10	-6
3,4,5,6-Tetrabromo-1,2-benzoquinone (TB-1,2-BQ)	424.7>	79.0	11.63±0.01	-70	-74	-10	-10
	424.7>	81.0^*^		-70	-74	-10	-10

DP: declustering potential; CE: collision energy; EP: entrance potential; CXP: cell exit potential; * quantitative ion.

## 2 结果与讨论

### 2.1 质谱条件的优化

使用甲醇(含0.25%甲酸)将HBQs混合标准中间液稀释至100 μg/L,使用针泵进样方式,分别在正离子模式和负离子模式下进行母离子扫描,以选择合适稳定的母离子。结果表明,13种HBQs在负离子模式下,以[M+H]^-^或M^-·^加合方式的母离子最为理想。选定母离子后对目标化合物进行子离子扫描,选择响应较强、干扰较少的两个子离子,进行碰撞能量(CE)和去簇电压(DP)的优化,优化后各目标化合物的质谱参数详见表1。

### 2.2 色谱条件的优化

#### 2.2.1 色谱柱的选择

根据目标化合物的性质,分别选择Waters ACQUITY UPLC BEH C_18_(100 mm×2.1 mm, 1.7 μm)和Waters ACQUITY UPLC HSS T_3_(100 mm×2.1 mm, 1.8 μm)两种通用型反相色谱柱进行考察。结果显示,HSS T_3_色谱柱可将所有目标化合物分离,与BEH C_18_色谱柱相比,除2,3-DBDMBQ外,其他目标化合物的峰形更加尖锐,灵敏度和信噪比更高且分离时间更短。因此,本实验选择HSS T_3_色谱柱。

#### 2.2.2 流动相的选择

甲酸可增加H^+^浓度,促进[M+H]^-^离子峰的形成^[20]^,但在负离子电离模式下也可抑制离子化效率。实验分别比较了不同体积分数(0%、0.1%、0.25%、0.5%)的甲酸水溶液和不同体积分数(0%、0.1%、0.25%、0.5%)的甲酸甲醇溶液作为流动相时目标化合物的响应和峰形。结果表明,以纯水和甲醇作为流动相时,虽然2,5-DCBQ、2,6-DCBQ、2,5-DBBQ、DCMBQ、TriCBQ、2,6-DIBQ、2,6-DBDMBQ、2,3-DBDMBQ的响应最强,但TCBQ、TBBQ、TC-1,2-BQ、TB-1,2-BQ的响应弱或不出峰。当水相中加入0.1%甲酸、有机相为甲醇时,13种HBQs整体响应较高,峰形最佳且稳定性最好。随着水相和有机相中甲酸体积分数增加,13种HBQs的响应强度逐渐降低。因此,选用0.1%甲酸水溶液-甲醇作为流动相。条件优化后13种HBQs的色谱图见图1,保留时间见表1。

**图1:**
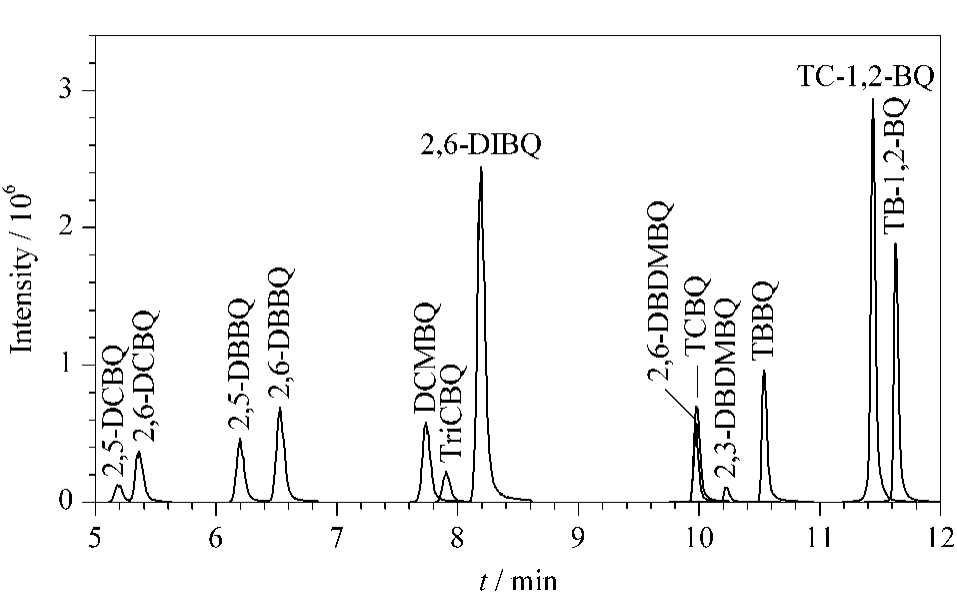
最优条件下13种HBQs的色谱图

### 2.3 前处理条件的优化

#### 2.3.1 固相萃取柱的选择

取水样500 mL,在20 ng/L加标水平下,根据目标物性质和填料成分,分别比较了Plexa柱(200 mg/6 mL,美国Agilent公司)、HLB柱(200 mg/6 mL,美国Waters公司)、ENV柱(200 mg/6 mL,美国Agilent公司)、MCX柱(150 mg/6 mL,美国Waters公司)、MAX柱(150 mg/6 mL,美国Waters公司)对水样中HBQs的萃取效果。计算各目标化合物的回收率,结果如图2所示。Plexa柱的萃取效果最佳,回收率最高。除TB-1,2-BQ外,其他12种目标物的回收率均高于70%。因此,选择Plexa固相萃取小柱对水样进行萃取富集。

**图2:**
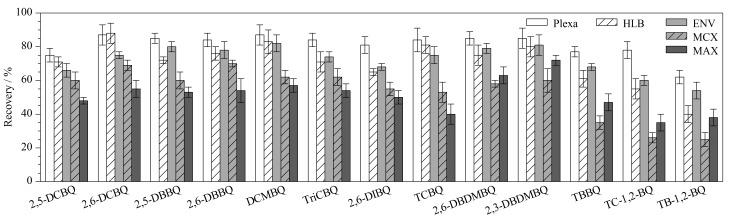
不同固相萃取柱对13种HBQs回收率的影响

#### 2.3.2 淋洗液的选择

上样后的淋洗步骤主要用于去除吸附在固相萃取柱上的干扰物。考虑到干扰物可能存在较宽的极性范围,实验采用两个淋洗步骤,第一步用含0.25%甲酸的水进行淋洗;第二步分别考察了不同体积分数(0、10%、20%、30%、40%、50%)甲醇水溶液(含0.25%甲酸)的淋洗效果。结果发现,当使用30%甲醇水溶液(含0.25%甲酸)时,目标化合物的回收率最高。当甲醇比例过高时,目标化合物也会被淋洗入废液中;当甲醇比例过低时,部分干扰物则会保留在柱上影响目标物的洗脱。因此,最终选择30%甲醇水溶液(含0.25%甲酸)作为第二步的淋洗液。

#### 2.3.3 氮吹温度的选择

为尽可能减少氮吹步骤引起的目标化合物损失,对比了不同氮吹温度(30、35、40、50 ℃)对HBQs回收率的影响。结果表明,目标化合物在30 ℃下氮吹的回收率最高。因此,将氮吹温度设定为30 ℃。

### 2.4 基质效应评价

质谱分析时样品中目标化合物以外的组分会影响其离子化过程,产生基质效应,导致目标化合物的信号相对于在纯溶剂中增强或减弱^[20⇓-22]^。用20%甲醇水溶液(含0.25%甲酸)稀释制备HBQs系列混合标准溶液,绘制溶剂标准曲线。用空白样品基质溶液稀释制备系列基质匹配混合标准溶液,绘制基质匹配工作曲线。采用基质匹配工作曲线和溶剂标准曲线斜率比来评价基质效应。当斜率比大于1时,为基质增强效应;当斜率比小于1时,为基质抑制效应;当斜率比为0.9~1.1时,基质效应可忽略^[20⇓-22]^。如表2所示,HBQs的基质效应为0.20~0.70, 13种HBQs均存在较强的基质抑制效应。因此,为有效消除基质效应带来的干扰,本研究使用基质匹配工作曲线进行定量分析。

**表2:** 饮用水中13种HBQs的线性关系、方法检出限、方法定量限和基质效应

Compound	Regression equation	r	Linear range/(μg/L)	MDL/(ng/L)	MQL/(ng/L)	Matrix effect
2,5-DCBQ	y=10138.9x+290.4	0.9996	0.5-100	0.5	1.7	0.70
2,6-DCBQ	y=23525.5x+1329.0	0.9993	0.5-100	0.3	0.9	0.61
2,5-DBBQ	y=26040.3x+1300.8	0.9998	0.5-100	0.8	2.5	0.58
2,6-DBBQ	y=30750.0x+1239.7	0.9998	0.5-100	0.5	1.7	0.51
DCMBQ	y=23013.0x-6502.2	0.9998	0.5-100	0.4	1.3	0.45
TriCBQ	y=8180.2x-2731.1	0.9994	1-100	1.5	4.6	0.40
2,6-DIBQ	y=75438.0x-15579.0	0.9996	0.5-100	0.2	0.6	0.39
TCBQ	y=12908.8x-6189.7	0.9999	2-100	1.7	5.7	0.22
2,6-DBDMBQ	y=9823.4x-1200.2	0.9997	2-100	1.5	5.0	0.24
2,3-DBDMBQ	y=620.21x-1062.8	0.9992	10-200	10.0	33.0	0.33
TBBQ	y=15687.3x-7489.5	0.9993	2-100	1.7	5.7	0.20
TC-1,2-BQ	y=75973.2x-40836	0.9998	0.5-100	0.3	0.9	0.42
TB-1,2-BQ	y=34732.9x-28283	0.9990	1-100	0.8	2.6	0.28

*y*: peak area; *x*: mass concentration, μg/L.

### 2.5 方法学考察

#### 2.5.1 线性范围、检出限和定量限

饮用水中13种HBQs的回归方程、相关系数和线性范围见表2。以目标化合物信噪比(*S/N*)为3时所对应的质量浓度为该化合物的检出限,*S/N*为10时所对应的质量浓度为定量限。结果显示,13种HBQs在各自的线性范围内均呈现良好的线性关系,相关系数均大于0.999。此方法具有较低的检出限和定量限,可满足饮用水中13种HBQs的分析检测。

#### 2.5.2 方法的准确度和精密度

按照所建立的实验方法,使用饮用水样品进行加标回收率测定。分别添加低(10 ng/L)、中(20 ng/L)、高(50 ng/L)3个水平的HBQs混合标准溶液,每个加标水平平行测定6次,计算不同加标水平下HBQs的回收率和相对标准偏差(RSD)。结果如表3所示,不同加标水平下13种HBQs的回收率为56%~88%, RSD为1.9%~9.2%,除TB-1,2-BQ外,其余HBQs的回收率均大于70%。该方法测定饮用水中HBQs含量的准确度和精密度均符合分析要求。

**表3:** 不同加标水平下13种HBQs的回收率和精密度(*n*=6)

Compound	10 ng/L			20 ng/L			50 ng/L	
	Recovery/%	RSD/%		Recovery/%	RSD/%		Recovery/%	RSD/%
2,5-DCBQ	77	4.8		75	3.7		73	7.1
2,6-DCBQ	85	3.0		87	4.8		81	3.6
2,5-DBBQ	85	4.8		83	2.7		80	1.9
2,6-DBBQ	87	4.2		82	4.0		85	2.6
DCMBQ	86	3.5		87	3.9		81	4.9
TriCBQ	83	2.8		84	3.4		78	5.7
2,6-DIBQ	78	7.0		81	5.4		77	3.7
TCBQ	80	3.7		84	3.0		79	2.6
2,6-DBDMBQ	85	3.3		88	2.3		84	4.4
2,3-DBDMBQ	84	1.9		85	3.7		85	1.9
TBBQ	82	3.3		77	6.2		75	4.6
TC-1,2-BQ	83	5.4		78	6.3		76	8.3
TB-1,2-BQ	66	6.6		61	7.4		56	9.2

### 2.6 方法对比

将本研究所建立方法与其他饮用水中HBQs的检测方法进行对比,如表4所示。本方法可同时检测饮用水中13种HBQs,检测种类多,且仪器分析时长较短,仅需16 min,检测效率较高。方法定量限处于较低水平,能够满足检测需求。已有研究采用Online SPE-LC-MS/MS对饮用水中的HBQs进行富集检测^[5]^,虽然所需水样体积较小,但部分目标物的定量限较高,且实验所用仪器较为昂贵,普及性不高。

**表4:** 本方法与其他方法的比较

Numbers of target compounds				Detection methods	Instrumental analysis time/min	MQL/(ng/L)		Ref.
Chloro- benzoquinones	Bromo- benzoquinones	Iodo- benzoquinone	Total					
6	6	1	13	SPE-UPLC-MS/MS	16	0.6	-33	this study
5	5	0	10	Online SPE-LC-MS/MS	/	0.2	-166	[5]
1	1	0	2	LLE-GC-ECD	>12	2.4	-2.7	[16]
1	0	0	1	SPE-HPLC-MS/MS	40	30		[23]
4	5	0	9	LLE-MicroLC-QTOF MS	20	1.9	-52.23	[24]

/: not mentioned; LLE: liquid-liquid extraction; ECD: electron capture detector; MicroLC-QTOF MS: micro fluid liquid chromatography and quadrupole-time of flight mass spectrometry.

### 2.7 实际水样的测定

使用本研究建立的方法,对5份末梢水进行检测。其中,2,6-DCBQ、2,5-DBBQ、2,6-DBBQ和2,6-DBDMBQ均有检出,检出率分别为100%、20%、80%和20%;各HBQs的检出含量分别为2,6-DCBQ 15.0~56.2 ng/L, 2,5-DBBQ 9.2 ng/L, 2,6-DBBQ 13.3~39.8 ng/L, 2,6-DBDMBQ 13.5 ng/L。实验结果说明,2,6-DCBQ与2,6-DBBQ检出较为普遍,检出含量也较高。

## 3 结论

本研究基于固相萃取-超高效液相色谱-三重四极杆质谱建立了饮用水中13种HBQs消毒副产物同时检测的分析方法。该方法高效简便,检测目标物的种类和数量较多,有机试剂消耗少,准确度和精密度均较高,适用于饮用水中HBQs的测定。应用该方法检测实际样品中的HBQs,发现饮用水中HBQs普遍存在。同时,该方法也为进一步研究饮用水中HBQs的分布特征、健康风险及控制措施奠定了基础。
